# circ-LIMK1 regulates cisplatin resistance in lung adenocarcinoma by targeting miR-512-5p/HMGA1 axis

**DOI:** 10.1515/med-2022-0542

**Published:** 2022-10-07

**Authors:** Ya Li, Fangfang Li, Yaya Wang, Fangyu Song, Lin Qi, Qiang Hu

**Affiliations:** Department of Respiratory and Critical Care Medicine, Xi’an Gaoling District Hospital, Xi’an, China; Department of Respiratory and Critical Care Medicine, The Third Affiliated Hospital of Xi’an Medical College, No. 277 Youyi West Road, Beilin District, Xi’an 710068, China

**Keywords:** circ-LIMK1, cisplatin, miR-512-5p, HMGA1, lung adenocarcinoma

## Abstract

This study aimed to unveil the detailed role and new mechanism of circ-LIMK1 in lung adenocarcinoma. Real-time quantitative polymerase chain reaction was performed to analyze the expression of circ-LIMK1, miR-512-5p, and HMGA1. 3-(4,5)-Dimethylthiahiazo(-*z-y1*)-3,5-di-phenytetrazoliumromide assay was employed to test the half maximal inhibitory concentration of cisplatin (DDP). Western blot was used to measure the expression of HMGA1, multidrug resistance protein 1, mitochondrial 37S ribosomal protein, and vascular endothelial growth factor A. Colony formation assay, flow cytometry, transwell assay, and tube formation assay were performed to analyze cell functions. Animal models were established to assay the role of circ-LIMK1 *in vivo*. The expression of circ-LIMK1 was up-regulated in DDP-resistant tumor tissues and cells. Knockdown of circ-LIMK1 reduced DDP resistance, impaired cancer cell growth, migration, invasion, and angiogenesis. circ-LIMK1 targeted miR-512-5p, and HMGA1 was targeted by miR-512-5p. MiR-512-5p absence could restore the repressive effects of circ-LIMK1 knockdown on lung adenocarcinoma cell phenotypes. Overexpression of HMGA1 could restore the inhibitory effects of miR-512-5p enrichment on lung adenocarcinoma cell malignant phenotypes. Knockdown of circ-LIMK1 could reduce growth of DDP-resistant tumors *in vivo*. Collectively, circ-LIMK1 regulated DDP resistance in lung adenocarcinoma by targeting miR-512-5p/HMGA1 axis.

## 1 Introduction

Lung adenocarcinoma, a subtype of non-small cell lung cancer (NSCLC), occupies about two-fifth of all lung cancers [1]. It is a cancer that develops because of abnormal and uncontrolled cell growth [2]. Smoking is the main cause of this cancer [3]. Besides, obesity and metabolic diseases (such as type 2 diabetes mellitus, dyslipidaemia, and cardiovascular diseases) may play a key role in increasing the risk of cancers, modulating pivotal cross-talk pathways for cancer cell proliferation and differentiation [4,5]. Recently, the treatment of lung adenocarcinoma has been very mature, but there are still poor prognosis and cisplatin (DDP) resistance, and the emergence of this reaction has become a new treatment challenge [6]. Therefore, it is essential to disclose lung adenocarcinoma pathogenesis to improve treatment outcomes.

As a kind of non-coding RNAs with closed-loop structures, circRNAs are ubiquitous in eukaryotes, and high abundance of circRNAs lays a foundation for their function [7]. In recent years, the role of circRNAs in human diseases has been discovered, but the functions of numerous novel circRNAs are still unknown [8,9]. The role of circRNAs in cancer-related processes is the most studied [10]. The functions of numerous circRNAs have been reported in lung adenocarcinoma [11,12]. For example, circ-104889 can regulate the invasion of lung adenocarcinoma cells by decoying miR-4458 [13]. However, there are few studies on the functional mechanism of circRNAs in lung adenocarcinoma. It has been reported that circ-LIMK1 (circ_0001715) might be a biomarker in lung adenocarcinoma [14]. So, this provide ideas for our research.

More and more studies have found that miRNAs have become key regulators in diverse biological processes [15,16]. These RNAs form complex networks that mediate cell differentiation, development, and homeostasis [17,18]. Because of this, miRNAs are involved in an increasing number of human disease regulation, especially in the development of cancer-related processes, including DDP chemoresistance [19–22]. MiR-512-5p has been extensively studied in NSCLC and can regulate lung cancer progression by targeting the expression of some genes [23–25]. However, the targeting relationship between miR-512-5p and circ-LIMK1 and its role on DDP resistance in lung adenocarcinoma are not clear.

High mobility group A1 (HMGA1) is active in a variety of tumors, and its biological function has been explored in many studies in recent years [26,27]. More recently, it has been found that HMGA1 can promote a variety of malignant phenotypes of cancer cells, and it has a high expression level in tumor tissues [28–30]. However, it has not been reported whether it is associated with adverse prognostic reactions and DDP resistance in the process of lung adenocarcinoma, and whether it is regulated by miR-512-5p.

In our study, the targeting relationship between miR-512-5p and circ-LIMK1 or HMGA1 and their functions in the progression of lung adenocarcinoma were confirmed, which provided new ideas for overcoming DDP resistance in lung adenocarcinoma in the future.

## 2 Methods

### 2.1 Patients sample collection

All tests were supported by the ethical committee of Xi’an Gaoling district hospital, and patient tissues were professionally identified and surgically removed in the hospital of Xi’an Gaoling district hospital. Patients have been informed of all trials and have signed informed consent forms. The clinical characteristics of patients are shown in Table 1. The tissues were immediately removed and stored in liquid nitrogen for temporary storage, and then transferred to −80°C.

**Table 1:** Clinical characteristics of lung adenocarcinoma patients according to high and low circ-LIMK1 expression

Clinicopathological features	Number of cases 56	circ-LIMK1 expression		*P*-value
		Low 28	High 28	
Age				
<55	19	10	9	0.778
≥55	37	18	19	
Gender				
Male	33	14	19	0.174
Female	23	14	9	
TNM stage				
I–II	20	15	5	0.005
III	36	13	23	
Lymph node metastasis				
Negative	23	18	5	<0.001
Positive	33	10	23	
Cisplatin therapy				
Chemosensitive	19	17	2	<0.001
Chemoresistant	37	11	26	

Chi-square test.

### 2.2 Cell culture and transfection

Human lung adenocarcinoma cell lines (A549 and H1975 cells) were purchased from American Type Culture Collection (ATCC, Rockville, MD, USA), normal epithelial cell line (16HBE) was bought from Chuan Qiu Biotechnology (Shanghai, China). Cells were cultured in a medium (RPMI-1640, Sigma-Aldrich, St. Louis, MO, USA) replenishing 10% fetal bovine serum (FBS, GIBCO, Carlsbad, CA, USA) with 1% penicillin streptomycin combination and then in a humid incubator containing 5% carbon dioxide at 37°C. DDP-resistant A549 and H1975 (A549/DDP and H1975/DDP) cells were triumphantly established by exposing A549 and H1975 cells to the accumulating DDP concentrations, until the ultimate density of DDP was 1 μg/mL.

Transfection experiments can begin after three generations of cell culture. The Lipofectamine 2000 transfection reagent (Invitrogen, Carlsbad, CA, USA) was adopted to transfect cells according to the operating instructions. Silencing of circ-LIMK1 (si-circ-LIMK1#1 or si-circ-LIMK1#2), miR-512-5p, pCD5-circ-LIMK1 (circ-LIMK1), miR-512-5p inhibitor (anti-miR-512-5p), pcDNA-HMGA1 (HMGA1), short hairpin (sh-circ-LIMK1), and negative controls (si-NC, miR-NC, Vector pCD5 [Vector]), miR-NC inhibitor (anti-miR-NC), pcDNA, sh-NC were synthesized and purchased from Ribobio (Guangzhou, China).

### 2.3 RNA extraction and real-time quantitative polymerase chain reaction (RT-qPCR)

Total RNA was isolated by Trizol reagent (TaKaRa, Tokyo, Japan). RNA concentrations and quality were detected with a NanoDrop 2000 spectrophotometer (Thermo Fisher Scientific, Waltham, MA, US). The AMV Reverse Transcriptase (Solarbio, Beijing, China) was adopted for reverse transcription of RNA into cDNA. SYBR Premix Ex Taq II (TaKaRa) was used for gene expression analysis. The results were collected and calculated using the 2^−ΔΔCt^ method. U6 and glyceraldehyde 3-phosphate dehydrogenase (GAPDH) were used as controls. The primers are shown in Table 2.

**Table 2:** The primer sequences for RT-qPCR

Name		Primer 5′-3′
circ-LIMK1	Forward	ATCGGGGTGCTCTACAAGGA
circ-LIMK1	Reverse	ACTACGCGGAGGGACTCAGA
LIMK1	Forward	ACAGAGGATGCTGTTGGCTT
LIMK1	Reverse	TAGTACTGGTGCGACAGGGA
miR-512-5p	Forward	GCCGAGCACTCAGCCTTGAG
miR-512-5p	Reverse	CTCAACTGGTGTCGTGGA
HMGA1	Forward	TTTTAAGCTCCCCTGAGCCG
HMGA1	Reverse	AATATTCCCCCTCCCCCGAA
GAPDH	Forward	AGCTCACTGGCATGGCCTTC
GAPDH	Reverse	CGCCTGCTTCACCACCTTCT
U6	Forward	CGCTTCACGAATTTGCGTGTCAT
U6	Reverse	GCTTCGGCAGCACATATACTAAAAT

### 2.4 RNase R digestion

RNA was extracted from the collected cells and digested with RNase R (10 U/μL, Beyotime, Shanghai, China) for 15 min. Then cDNA was synthesized, and RT-qPCR analysis was performed to verify the stability of circ-LIMK1.

### 2.5 3-(4,5)-Dimethylthiahiazo(-*z-y1*)-3,5-di-phenytetrazoliumromide (MTT) assay

Cell viability and cell sensitivity were determined by MTT assay. The transfected cells were transferred to 96-well plates for further culture, and then 10 μL MTT (Beyotime) was added into each well, and the half-maximal inhibitory concentration (IC_50_) value and cell viability of DDP-resistant cells were measured after 4 h incubation in the incubator.

### 2.6 Western blot

The transfected cells were washed and collected and a protein extraction kit (Beyotime) was used on ice to lyse the cells, followed by centrifugation to collect the supernatant. The protein samples were treated at high temperature, and then 10% SDS-PAGE was used for protein isolation. The protein was then used for transferring to the PVDF membrane (Millipore, Billerica, MA, USA). The PVDF membrane was then sealed with 5% BSA, and was incubated with primary antibodies and second antibody successively. Finally, chemiluminescence reagent (ECL, Beyotime) was applied to develop the PVDF membrane. Primary and secondary antibodies were bought from Abcam (Cambridge, UK), including multidrug resistance mutation (MDR1, 1:1,000, ab170904), multidrug resistance-associated protein 1 (MRP1, 1:1,000, ab233383), GAPDH (1:2,000, ab8245), anti-vascular endothelial growth factor A (VEGFA, 1:1,000, ab52917), anti-HMGA1 (1:1,000, ab129153), anti-β-actin (1:2,000, ab179467), and horse radish peroxidase-conjugated secondary antibody (1:5,000, ab288151).

### 2.7 Colony formation assay

After transfected cells were digested by using trypsin, the cells were prepared into suspension cells, and then added to the culture plate. When cells were cultured for 2 weeks, the cell culture was terminated. Subsequently, 4% paraformaldehyde (Solarbio) was employed to fix the cells and 1% of crystal violet stain (Solarbio) was used to stain the cells. Finally, the cells were observed and photographed under a microscope.

### 2.8 Cell apoptosis analysis

Annexin V-FITC and PI were adopted to stain the apoptotic cells and cells were distinguished using flow cytometry (Beckman Coulter, Kraemer Boulevard, CA, USA). First, the transfected cells were prepared into cell suspension, and then 100 μL cell suspension was added to the tube. Next, the Annexin V-FITC and PI was appended to stain the cells, which was gently mixed and placed in dark for 15 min. Annexin V/PI cell apoptosis analysis kit (Servicebio) was performed to measure the cell apoptosis based on the product’s instruction.

### 2.9 Transwell assay

Experiment was conducted according to Transwell (Corning Life Sciences, Corning, NY, USA) instructions. Transwell cells were first placed into the culture plate, and then transfected cells were prepared into cell suspension. Suspended cells were added into the upper chamber, and DMEM medium replenishing 10% FBS was added into the lower chamber to detect the migration of cells from the upper chamber to the lower chamber. The cell invasion assay separated the upper compartment from the lower compartment by a membrane containing Matrigel matrix (BD Biosciences, San Jose, CA, USA), and then measured the invasion of the upper compartment into the lower compartment after digesting the matrix by using microscope.

### 2.10 Tube formation assay

The thawed extracellular matrix was first added to the pre-cooled 96-well plate, and then incubated at 37°C for 1 h. Subsequently, the transfected cell suspension prepared in advance was inoculated into a 96-well plate according to a certain density for further culture for 18 h. Finally, the culture medium was discarded to clean the well, and after staining and incubation, fluorescence microscope was used for photo analysis.

### 2.11 Dual-luciferase reporter assay

The luciferase reporter vectors of wild type circ-LIMK1 (wt-circ-LIMK1) and mutant circ-LIMK1 (mut-circ_LIMK1), wt-HGMA1 3′UTR, and mut-HGMA1 3′UTR were bought from Hanbio Biotechnology (Shanghai, China). Luciferase Activity Determination Kit was purchased from Beyotime. After transfection, cells were lysed with luciferase lysis solution. After cell lysis supernatant was collected, 50 μL supernatant was taken and added to the 96-well plate, and then 100 μL luciferase detection reagent was added to the 96-well plate under fast light protection. After shaking and mixing in the tester, luciferase activity was measured, and data were recorded and analyzed.

### 2.12 RNA immunoprecipitation (RIP) assay

EZMagna RIP kit used for RIP assay was purchased from Millipore. After the transfected cells were cleaned with PBS, the cells were scraped off with cell wiper and collected into the centrifuge tube. After collecting the cells, the cells were re-suspended with RIP lysis solution of the same volume as the cells. The magnetic beads (Abcam) were re-suspended, 50 μL of them were absorbed and put into each EP tube. RIP Wash Buffer was appended for vortex shock, and 100 μL RIP Wash Buffer was added after three times of cleaning to re-suspend the magnetic beads. Anti-lgG or anti-Ago2 (Beyotime) were added and then incubated at 25°C for 1 h. The cell lysate was hatched with the magnetic bead–antibody complex and hatched at 4°C overnight. Then the magnetic beads were separated by high-speed centrifugation, and the supernatant was collected for RNA purification, followed by subsequent analysis.

### 2.13 Tumor formation assay

H1975/DDP (1 × 10^7^) cells infected with lentivirus-mediated sh-NC or sh-circ-LIMK1 were injected subcutaneously into nude mice (*n* = 5/group, Vital River Laboratory Animal Technology, Beijing, China). After 7 days, the mice were injected with DDP (5 mg/kg) or equivalent PBS every 5 days, and the tumor volume (volume (mm^3^) = width^2^ × length/2) was determined. After 32 days, the mice were euthanized to determine the tumor weight. The expression of three tumor factors was determined, and the expression of Ki67 and HMGA1 was determined by immunohistochemistry (IHC). All animal experiments are supported and approved by the Xi’an Gaoling district hospital Animal Ethics Committee.

### 2.14 IHC assay

First, the fresh tissues were sliced and grilled, then dewaxed and repaired. It was followed by containment, and then incubated with primary antibodies (Ki67, 1:100, ab15580 and HMGA1, 1:100, ab129153, Abcam) and secondary antibody, successively. Then DAB chromogenic reagent (Beyotime) was used to react for 15 min, and hematoxylin was used to stain for 5 min. After the excess staining solution was removed, the tablets were sealed for microscopic examination.

### 2.15 Statistical analysis

Statistical analysis was employed by using GaphPad Prism 7.0, and the data were measured as mean ± standard deviation (SD). *P* < 0.05 represented statistically significant. Comparisons between two groups or among multiple groups were tested by using student’s *t*-test or one-way analysis of variance. There were three parallel experiments in each group. Pearson correlation analysis was adopted to analyze the correlation.


**Ethics approval and consent to participate:** The present study was approved by the ethical review committee of Xi’an Gaoling district hospital. Written informed consent was obtained from all enrolled patients.
**Consent for publication:** Patients agreed to participate in this work.

## 3 Results

### 3.1 The expression of circ-LIMK1 was significantly up-regulated in DDP-resistant lung adenocarcinoma tissues and cells

**Figure 1:**
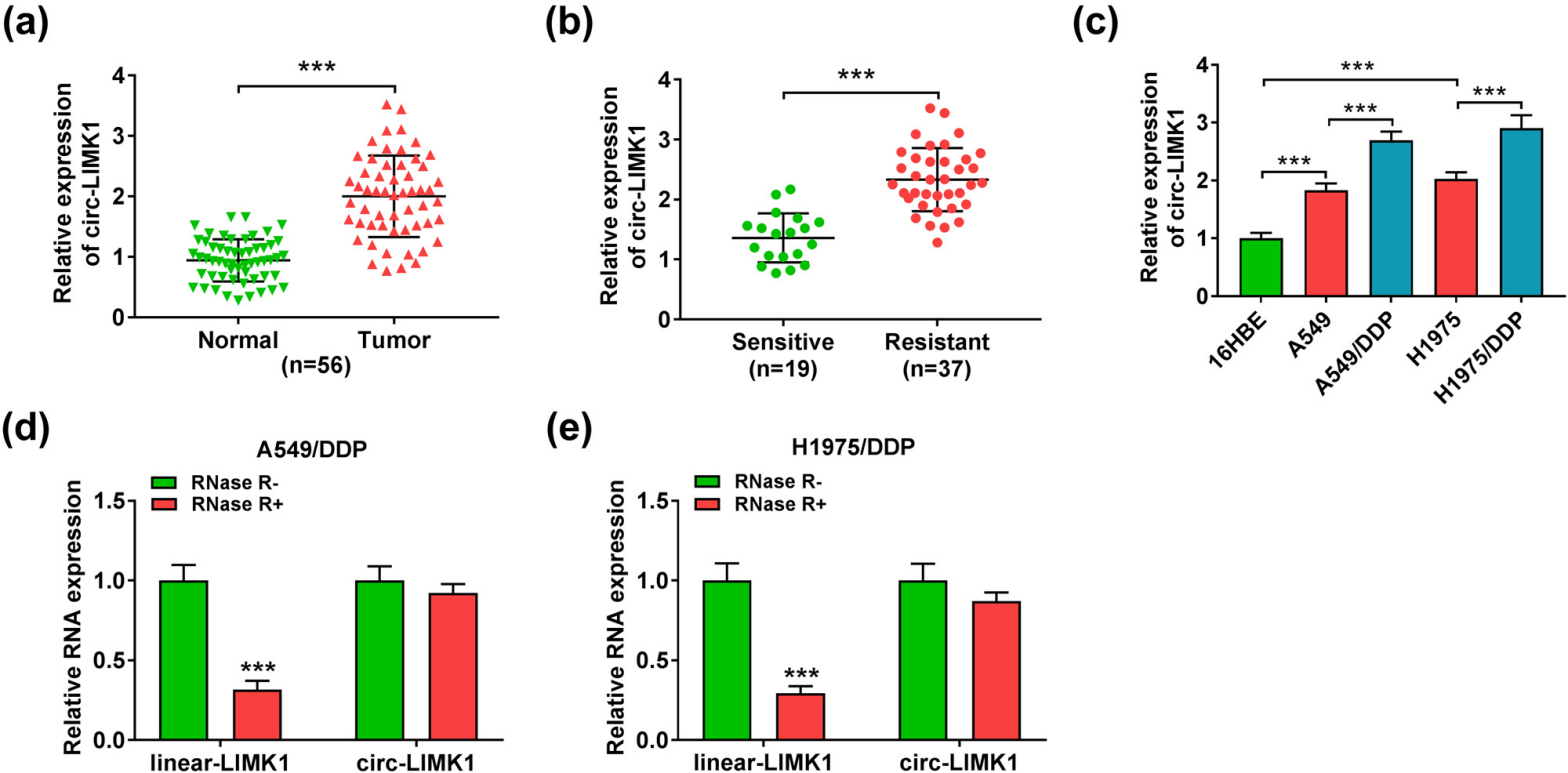
circ-LIMK1 was highly expressed in DDP-resistant tumor tissues and cells. (a) RT-qPCR was used to analyze the expression of circ-LIMK1 in normal tissues and tumor tissues. (b) RT-qPCR was performed to detect the expression of circ-LIMK1 in sensitive tumor tissues and resistant tumor tissues. (c) RT-qPCR was employed to analyze the expression of circ-LIMK1 in 16HBE, A549, A549/DDP, H1975, and H1975/DDP cells. (d and e) RT-qPCR was used to measure the expression of circ-LIMK1 and linear LIMK1 in A549/DDP and H1975/DDP cells after RNase R digestion. *P < 0.05, ** *P* < 0.01, ***P < 0.001.

Lung cancer is one of the most difficult cancers to treat in the world today. Its poor prognosis and the emergence of DDP resistance bring great challenges to its treatment [31]. Therefore, to further explore the relevant mechanisms of lung cancer, we conducted this study. In the beginning, the results of RT-qPCR analysis proved that circ-LIMK1 was raised in tumor tissues (*n* = 56/group) than that in normal tissues (Figure 1a). Second, circ-LIMK1 was analyzed by RT-qPCR, the results suggested that the expression of circ-LIMK1 was boosted in resistant tissues (*n* = 37) compared with circ-LIMK1 in sensitive tissues (*n* = 19) (Figure 1b). Meanwhile, it was found that circ-LIMK1 was significantly correlated with the stage of lung adenocarcinoma patients and other clinical characteristics such as DDP resistance (Table 1). Besides, the results presented that the expression of circ-LIMK1 was up-regulated in A549, A549/DDP, H1975, and H1975/DDP cells, then it was more markedly up-regulated in A549/DDP and H1975/DDP cells (Figure 1c). These results preliminarily confirmed the higher level of circ-LIMK1 expression in DDP-resistant cells of lung adenocarcinoma. Our study also found that the expression of circ-LIMK1 had no difference, but linear LIMK1 was reduced in A549/DDP and H1975/DDP cells after digested by RNase R (Figure 1d and e). In a word, the expression of circ-LIMK1 was at a higher level in DDP-resistant tissues and in cells of lung adenocarcinoma.

### 3.2 Knockdown of circ-LIMK1 could reduce the resistance of DDP-resistant lung adenocarcinoma cells

**Figure 2:**
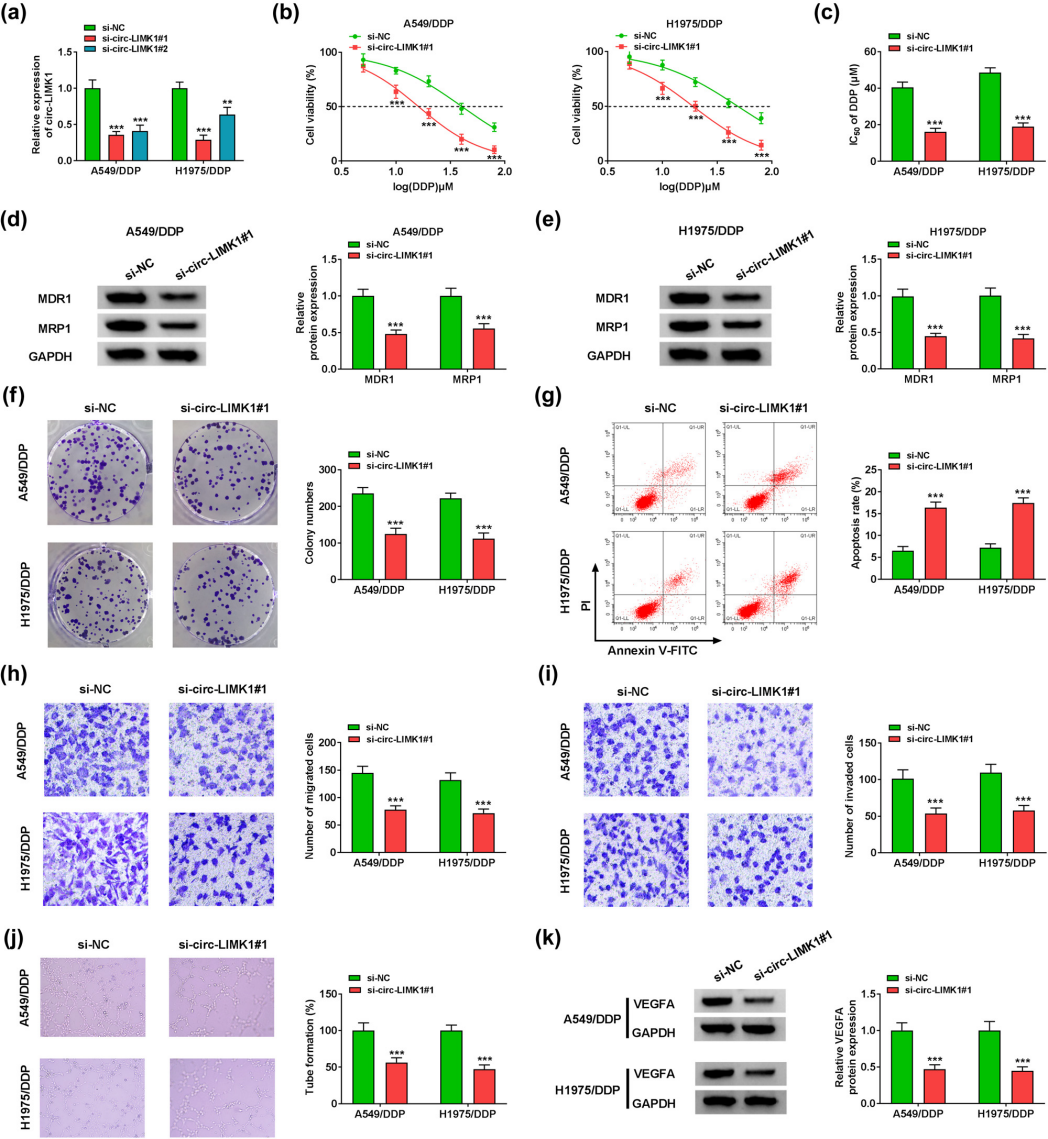
Knockdown circ-LIMK1 could influence cell proliferation, migration, invasion, DDP resistance, tube formation, and apoptosis in lung adenocarcinoma DDP-resistant cells. (a) RT-qPCR was applied to examine the expression of circ-LIMK1 in A549/DDP and H1975/DDP cells after transfected with si-NC, si-circ-LIMK1#1, and si-circ-LIMK1#2. A549/DDP and H1975/DDP cells were transfected with si-NC or si-circ-LIMK1#1. (b) MTT assay was used to analyze cell viability. (c) MTT assay was applied to detect IC_50_ value. (d and e) Western blot was used to measure the protein levels of MDR1 and MRP1. (f) Colony formation assay was employed to analyze the number of colonies. (g) Flow cytometry was applied to detect cell apoptosis. (h and i) Transwell assay was employed to test cell migration and invasion. (j) Tube formation assay was used to analyze the tube formation. (k) Western blot was adopted to measure the expression of VEGFA. *P < 0.05, ** P < 0.01, ***P < 0.001.

The expression of circ-LIMK1 had been detected in our experiments, then the specific role of circ-LIMK1 in DDP-resistant lung adenocarcinoma cells needs to be further explored. Initially, we analyzed the knockdown effect of circ-LIMK1, which indicated that the knockdown effect of si-circ-LIMK1#1 was more significant than that of si-circ-LIMK1#2 in A549/DDP and H1975/DDP cells (Figure 2a). Therefore, si-circ-LIMK1#1 was selected for subsequent experimental study. MTT assay demonstrated that the IC_50_ value of DDP was decreased after transfected with si-circ-LIMK1#1 in A549/DDP and H1975/DDP cells (Figure 2b and c), indicating that circ-LIMK1 knockdown enhanced DDP sensitivity in DDP-resistant lung adenocarcinoma cells. Moreover, western blot analysis confirmed that the DDP-resistant related protein levels of MDR1 and MRP1 were reduced in A549/DDP and H1975/DDP cells after circ-LIMK1 knockdown compared with that in negative control (Figure 2d and e). Beyond that, colony formation assay proved that colonies number was lower in A549/DDP and H1975/DDP cells after circ-LIMK1 knockdown (Figure 2f). The statistic results of cell apoptosis by flow cytometry suggested that A549/DDP and H1975/DDP cell apoptosis was enhanced after knockdown of circ-LIMK1 (Figure 2g). The migration and invasive abilities were determined by transwell assays, these results demonstrated that knockdown of circ-LIMK1 could restrain A549/DDP and H1975/DDP cell migration and invasion (Figure 2h and i). At the same time, the results of the tube formation assay analysis confirmed that knockdown of circ-LIMK1 could suppress the number of tube formation (Figure 2j). In the end, western blot was performed to analyze the related protein level of tube formation expression in A549/DDP and H1975/DDP cells, the results exhibited that circ-LIMK1 knockdown could reduce the protein of VEGFA level in A549/DDP and H1975/DDP cells (Figure 2k), it meant that the number of tubes was reduced after knockdown of circ-LIMK1. In summary, circ-LIMK1 knockdown could inhibit cell proliferation, migration, invasion, and tube formation, and also could promote cell apoptosis in A549/DDP and H1975/DDP cells.

### 3.3 circ-LIMK1 could target miR-512-5p in DDP resistance of lung adenocarcinoma cells

**Figure 3:**
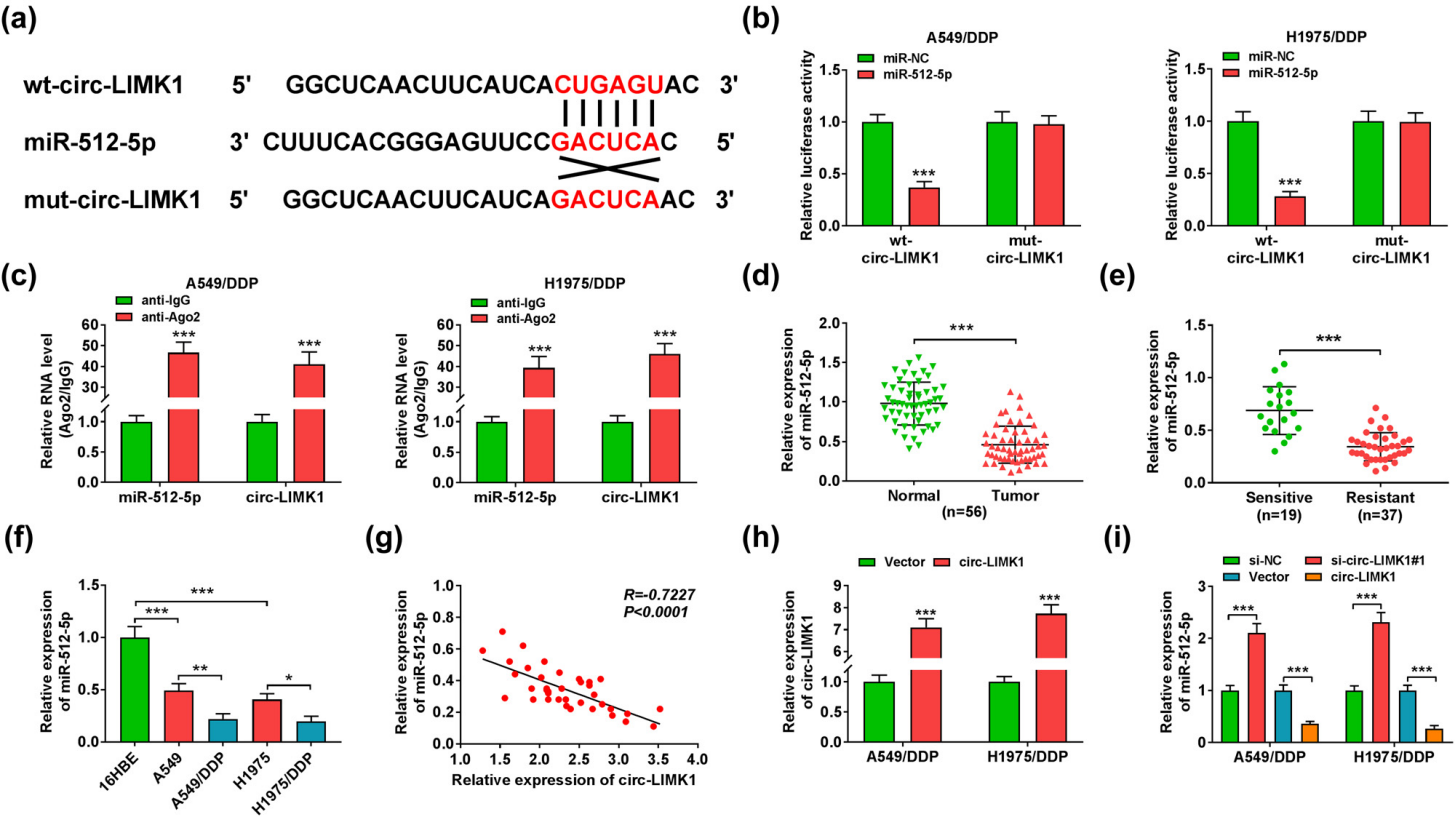
circ-LIMK1 could target miR-512-5p in lung adenocarcinoma DDP-resistant cells. (a) Circular RNA interactome predicted the targeted binding sites of miR-512-5p and circ-LIMK1. (b) The luciferase activities of wt-circ-LIMK1 or mut-circ-LIMK1 were analyzed by dual-luciferase reporter assay in A549/DDP and H1975/DDP cells after transfected with miR-NC or miR-512-5p. (c) The target relationship between miR-512-5p and circ-LIMK1was measured by RIP assay. (d) RT-qPCR was performed to test the expression of miR-512-5p in normal tissues and tumor tissues. (e) The expression of miR-512-5p in sensitive tumor tissues and resistant tumor tissues was detected by RT-qPCR. (f) RT-qPCR was employed to measure the expression of miR-512-5p in 16HBE, A549, A549/DDP, H1975, and H1975/DDP cells. (g) Pearson correlation analysis was used to detect the correlation between miR-512-5p and circ-LIMK1. (h) RT-qPCR was performed to measure the expression of circ-LIMK1 in A549/DDP and H1975/DDP cells after transfected with vector or circ-LIMK1. (i) RT-qPCR was employed to test the expression of circ-LIMK1 in A549/DDP and H1975/DDP cells after transfected with si-NC, si-circ-LIMK1#1, vector, or circ-LIMK1. *P < 0.05, ** *P* < 0.01, ***P < 0.001.

It has been reported in previous studies that miR-512-5p expression was down-regulated in small lung cancer cells and inhibited the proliferation of tumor cells [25]. Therefore, we also investigated it. First, circular RNA interactome (https://circinteractome.nia.nih.gov/) prediction revealed the existence of targeted binding sites between circ-LIMK1 and miR-512-5p (Figure 3a). The luciferase activity of circ-LIMK1 detected by dual-luciferase reporter assay showed that the luciferase activity of wild type circ-LIMK1 (wt-circ-LIMK1) was significantly decreased under the condition of miR-512-5p overexpression, while there was no significant difference in luciferase activity of mutant circ-LIMK1 (mut-circ-LIMK1) in A549/DDP and H1975/DDP cells (Figure 3b). Meanwhile, RIP assay revealed that there was a direct interaction between miR-512-5p and circ-LIMK1 in A549/DDP and H1975/DDP cells (Figure 3c). After that, using RT-qPCR to analyze miR-512-5p expression, the results presented that miR-512-5p was lower in tumor tissues (*n* = 56) than that in normal tissues (Figure 3d), the expression of miR-512-5p was down-regulated in resistant tumor tissues (*n* = 37) compared with miR-512-5p in sensitive tumor tissues (*n* = 19) (Figure 3e). The expression of miR-512-5p in 16HBE, A549, A549/DDP, H1975, and H1975/DDP cells was also analyzed by the method of RT-qPCR, the results proved that the expression of miR-512-5p was down-regulated in tumor cell lines (A549, A549/DDP, H1975, and H1975/DDP), and the expression of miR-512-5p was prominently lower in A549/DDP and H1975/DDP cells (Figure 3f). Pearson correlation analysis disclosed that there was a negative correlation between miR-512-5p and circ-LIMK1 (Figure 3g). We detected the expression of circ-LIMK1 under transfection of circ-LIMK1 or Vector condition, and the results showed that circ_LIMK1 overexpression made circ-LIMK1 expression up-regulate (Figure 3h). Detection of miR-512-5p expression results illustrated that circ-LIMK1 knockdown could increase the expression of miR-512-5p, then miR-512-5p was inhibited after transfected with circ-LIMK1 (Figure 3i). In short, there was a target relationship between circ-LIMK1 and miR-512-5p.

### 3.4 Anti-miR-512-5p co-transfection could restore the effect of si-circ-LIMK1#1 transfection in the related progress of DDP-resistant lung adenocarcinoma cells

**Figure 4:**
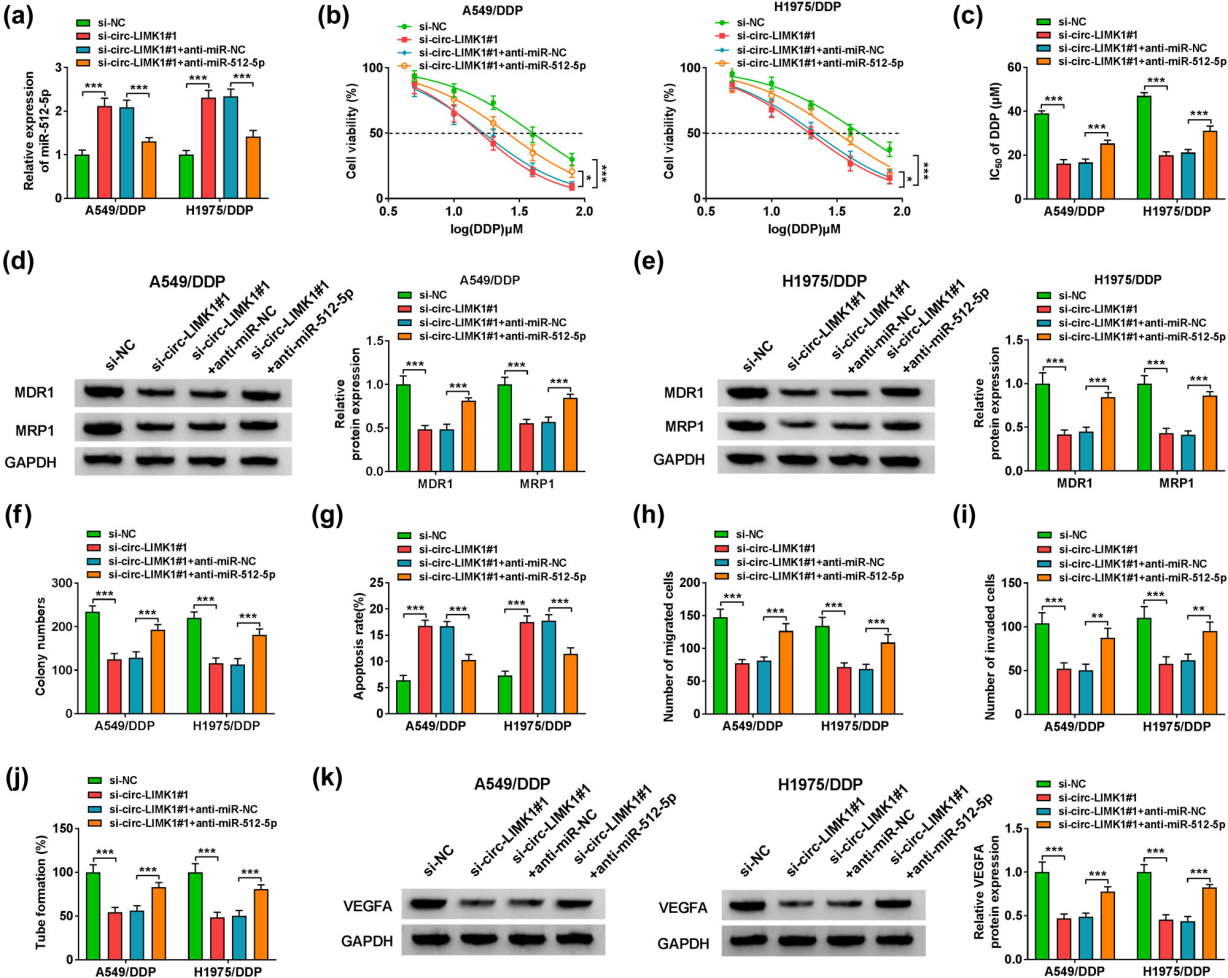
MiR-512-5p could overturn the effect of si-circ-LIMK1 in the progress of lung adenocarcinoma DDP-resistant cells. A549/DDP and H1975/DDP cells were transfected with si-NC, si-circ-LIMK1#1, si-circ-LIMK1#1 + anti-miR-NC, or si-circ-LIMK1#1 + anti-miR-512-5p. (a) The expression of miR-512-5p by RT-qPCR. (b) MTT assay was used to measure cell viability. (c) The IC_50_ value of A549/DDP and H1975/DDP cells was measured by using MTT assay. (d and e) Western blot was used to measure the expression of MDR1 and MRP1. (f) The number of colonies was measured by colony formation assay. (g) Cell apoptosis was detected by flow cytometry. (h and i) Cell migration and invasion was tested by transwell assay. (j) Tube formation assay was employed to analyze tube formation. (k) Western blot was adopted to detect the expression of VEGFA. *P < 0.05, ** *P* < 0.01, ***P < 0.001.

The role of miR-512-5p in the related process of lung adenocarcinoma needs to be studied timely, so we conducted the following experiments. First, we analyzed the expression of miR-512-5p, which showed that circ-LIMK1 knockdown could promote miR-512-5p expression, but miR-512-5p inhibitor could weaken the effect of knockdown of circ-LIMK1 on miR-512-5p expression in A549/DDP and H1975/DDP cells (Figure 4a). Moreover, MTT assay results disclosed that knockdown of circ-LIMK1 decreased the IC_50_ value of DDP in A549/DDP and H1975/DDP cells; however, the IC_50_ value of DDP in A549/DDP and H1975/DDP cells was subverted after co-transfected with miR-512-5p inhibitor (Figure 4b and c). Besides, the results of western blot proved that circ-LIMK1 knockdown could reduce the protein of MDR1 and MRP1 expression, and then co-transfection with miR-512-5p could partly restore the expression of MDR1 and MRP1 in A549/DDP and H1975/DDP cells (Figure 4d and e). The colony formation assay was utilized to examine the proliferative potential of cells, and the results also showed that the number of colonies was decreased in A549/DDP and H1975/DDP cells after knockdown of circ-LIMK1, but miR-512-5p silencing could overturn the tendency, so anti-miR-512-5p co-transfection could reverse the effect of si-circ-LIMK1#1 transfection in cell proliferation (Figure 4f). In addition, the detection of cell apoptosis via flow cytometry displayed that circ-LIMK1 knockdown could facilitate A549/DDP and H1975/DDP cells apoptosis; however, miR-512-5p reducing could reduce the influence of circ-LIMK1 silencing on cell apoptosis (Figure 4g). According to the transwell assay, this study counted the migratory and invasive cells in the transwell chambers; the results disclosed that circ-LIMK1 knockdown inhibited A549/DDP and H1975/DDP cell migration and invasion, then cell migration and invasion were recovered after co-transfected with miR-512-5p inhibitor (Figure 4h and i). Then tube formation assay analyzed the number of tubes, and the results presented that knockdown of circ-LIMK1 could restrain tubes formation, but miR-512-5p down-regulation could overthrow the trend (Figure 4j). Last but not the least, western blot analyzed the tubes related protein VEGFA and revealed that knockdown of circ-LIMK1 lessened the expression of VEGFA in A549/DDP and H1975/DDP cells, then co-transfected with anti-miR-512-5p could weaken the effect of circ-LIMK1 decreasing the expression of VEGFA (Figure 4k). In short, miR-512-5p inhibitory could overturn the effect of si-circ-LIMK1#1 transfection on cell proliferation, migration, invasion, apoptosis, tube formation, and DDP resistance in A549/DDP and H1975/DDP cells.

### 3.5 There was a targeting relationship between miR-512-5p and HMGA1 in DDP-resistant lung adenocarcinoma cells

**Figure 5:**
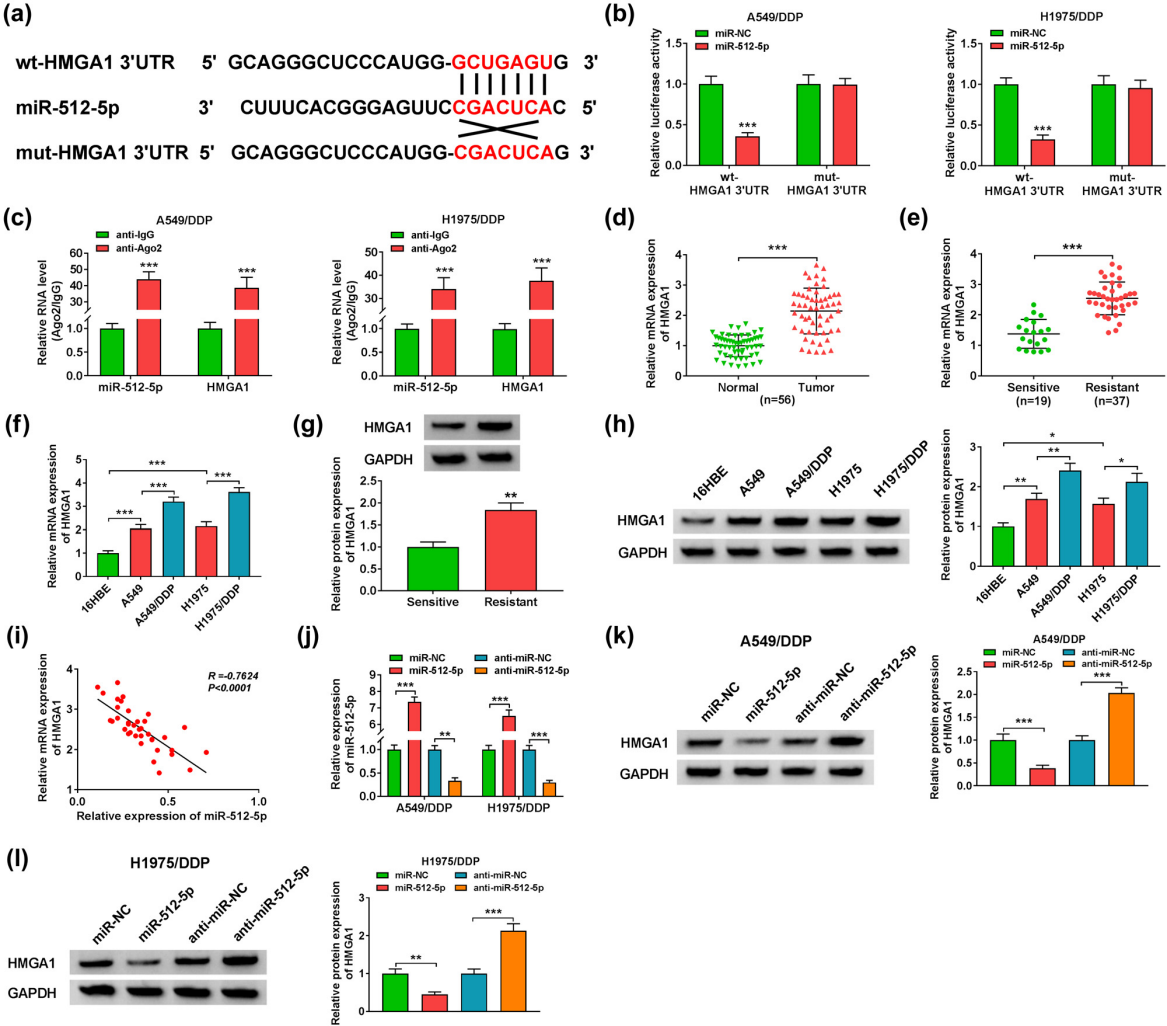
The target relationship between miR-512-5p and HMGA1 in lung adenocarcinoma DDP-resistant cells. (a) Target Scan Human7.2 predicted the targeted binding sites of miR-512-5p to HMGA1. (b) The luciferase activities of wt-HMGA1 3′UTR and mut-HMGA1 3′UTR were detected by using dual-luciferase reporter assay after transfected with miR-NC or miR-512-5p. (c) The interaction between miR-512-5p and HMGA1 was analyzed by using RIP assay after transfected with miR-NC or miR-512-5p. (d) RT-qPCR was applied to measure the expression of HMGA1 in normal tissues and tumor tissues. (e) RT-qPCR was performed to analyze the expression of HMGA1 in sensitive tumor tissues and resistant tumor tissues. (f) The expression of HMGA1 in 16HBE, A549, A549/DDP, H1975, and H1975/DDP cells was tested by RT-qPCR. (g) The protein of HMGA1 in sensitive tumor tissues and resistant tumor tissues was measured by western blot. (h) The protein level of HMGA1 in 16HBE, A549, A549/DDP, H1975, and H1975/DDP cells was examined by western blot. (i) Pearson correlation analysis was used to analyze the correlation between miR-512-5p and HMGA1. (j) RT-qPCR was applied to measure the expression of miR-512-5p in A549/DDP and H1975/DDP cells after transfected with miR-NC, miR-512-5p, anti-miR-NC, or anti-miR-512-5p. (k and l) Western blot was performed to test the protein level of HMGA1 in A549/DDP and H1975/DDP cells after transfected with miR-NC, miR-512-5p, anti-miR-NC, or anti-miR-512-5p. *P < 0.05, ** *P* < 0.01, ***P < 0.001.

Some studies have found that HMGA1 plays an important role in the study of lung adenocarcinoma-related processes [29], but the relationship between HMGA1 and miR-512-5p has not been reported. Therefore, we have carried out a series of studies to investigate the relationship between the two. First of all, as shown in Figure 5a, Target Scan Human7.2 (http://www.targetscan.org/vert_72/) predicted that miR-512-5p and HMGA1 had targeted binding sites. Further verification showed that miR-512-5p could reduce the luciferase activity of wild-type HMGA1 (wt-HMGA1 3′UTR), while there was no significant difference in the luciferase activity of mutant HMGA1 (mut-HMGA1 3′UTR), which preliminarily confirmed the targeted binding of miR-512-5p and HMGA1 (Figure 5b). RIP experiment further confirmed that miR-512-5p could directly target HMGA1 (Figure 5c). In addition, RT-qPCR analysis of the RNA level of HMGA1 showed that HMGA1 expression was up-regulated in tumor tissues compared with normal tissues (*n* = 56/group) (Figure 5d), the expression of HMGA1 was boosted in resistant tumor tissues (*n* = 37) compared with sensitive tumor tissues (*n* = 19) (Figure 5e). Meanwhile, RT-qPCR for the analysis of the mRNA level of HMGA1 in A549, A549/DDP, H1975, and H1975/DDP cells was conducted, the results suggested that the mRNA level of HMGA1 was up-regulated, and HMGA1 was higher in A549/DDP and H1975/DDP cells than in the expression of HMGA1 in A549 and H1975 cells (Figure 5f). The protein expression of HMGA1 was increased in resistant tumor tissues compared with HMGA1 in sensitive lung adenocarcinoma tumor tissues (Figure 5g). Data on western blot analysis of the protein of HMGA1 suggested that HMGA1 protein was up-regulated in A549, A549/DDP, H1975, and H1975/DDP cells, while level of HMGA1 was markedly boosted in A549/DDP and H1975/DDP cells (Figure 5h). Pearson correlation analysis showed that there was a negative correlation between miR-512-5p and HMGA1 (Figure 5i). However, analysis of miR-512-5p expression in A549/DDP and H1975/DDP cells revealed that the expression of miR-512-5p could be raised after transfected with miR-512-5p, but miR-512-5p production was down-regulated after transfected with anti-miR-512-5p (Figure 5j). In the meantime, the protein level of HMGA1 was detected in A549/DDP and H1975/DDP cells, and demonstrated that miR-512-5p could inhibit the expression of HMGA1 but anti-miR-512-5p transfection could promote the expression of HMGA1 (Figure 5k and l). In a word, miR-512-5p could target HMGA1 in lung adenocarcinoma cells.

### 3.6 Overexpression of HMGA1 weakened the effect of miR-512-5p on the progression of DDP-resistant lung adenocarcinoma cells

**Figure 6:**
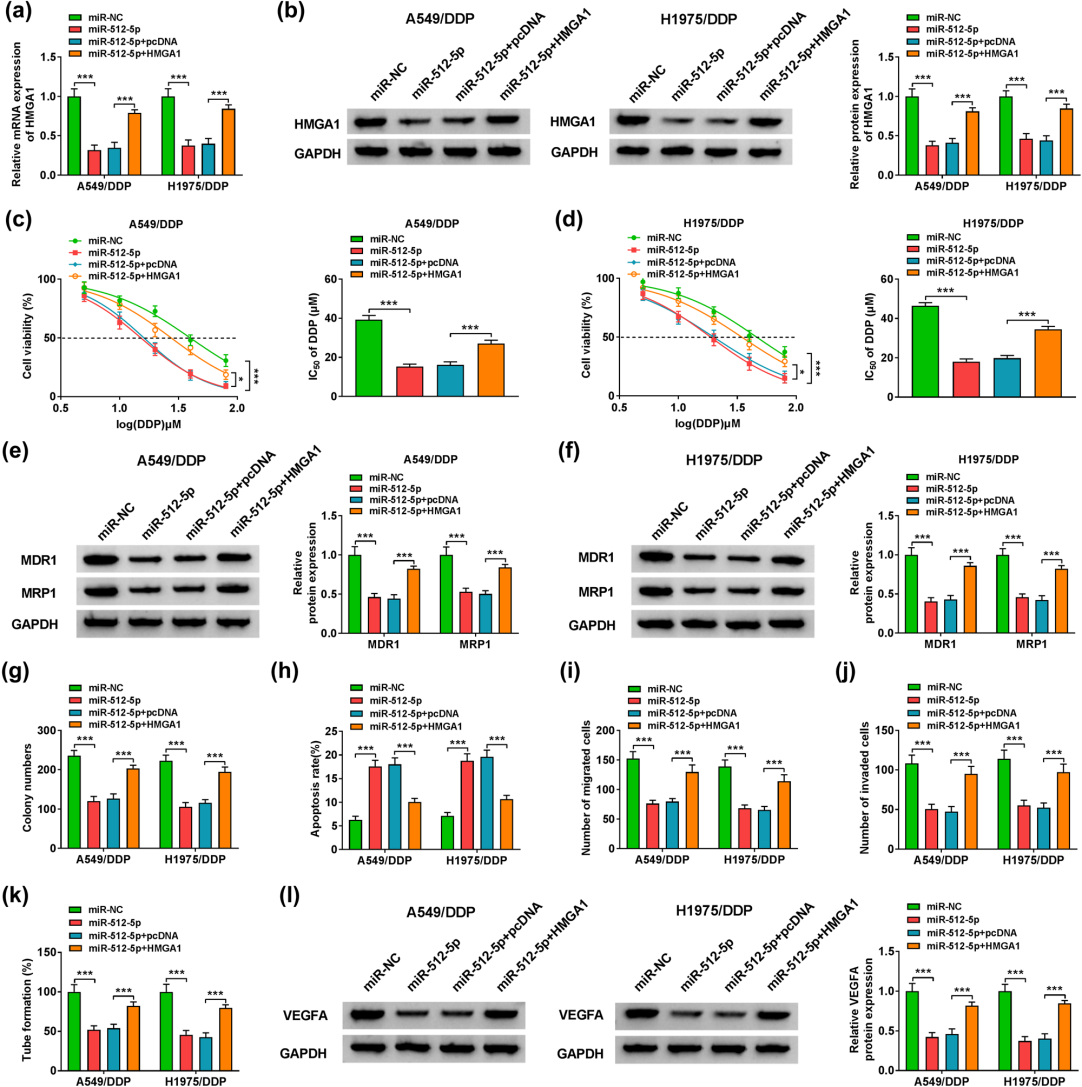
Overexpression HMGA1 could reverse the influence of miR-512-5p in lung adenocarcinoma DDP-resistant cells. A549/DDP and H1975/DDP cells were transfected with miR-NC, miR-512-5p, miR-512-5p + pcDNA, or miR-512-5p + HMGA1. (a) The mRNA level of HMGA1 was analyzed by RT-qPCR. (b) The protein level of HMGA1 was detected by western blot. (c and d) Cell viability and IC_50_ value were measured by MTT assay. (e and f) The protein levels of MDR1 and MRP1 were analyzed by western blot. (g) The number of colonies were analyzed by colony formation assay. (h) Flow cytometry was used to detect cell apoptosis. (i and j) Cell migration and invasion were analyzed by transwell assay. (k) Tube formation was analyzed by tube formation assay. (l) Western blot was performed to analyze the protein level of VEGFA. *P < 0.05, ** *P* < 0.01, ***P < 0.001.

To begin with, HMGA1 expression was analyzed by RT-qPCR to detect the level of HMGA1 mRNA and by western blot to examine the HMGA1 protein level, and the results showed that the expression of HMGA1 was down-regulated after transfected with miR-512-5p, but HMGA1 overexpression restored HMGA1 level in A549/DDP and H1975/DDP cells (Figure 6a and b). Second, MTT assay analysis confirmed that overexpression of HMGA1 could overturn the restrain effect of miR-512-5p on A549/DDP and H1975/DDP cells IC_50_ value of DDP (Figure 6c and d). At the same time, we tested the DDP resistance related protein levels of MDR1 and MRP1 in A549/DDP and H1975/DDP cells, the results demonstrated that the expression levels of MDR1 and MRP1 were lower after transfected with miR-512-5p, but the expression of MDR1 and MRP1 was restored after overexpression of HMGA1 (Figure 6e and f). Moreover, colony formation assay also showed that the number of cell colonies was decreased after miR-512-5p transfection; however, HMGA1 overexpression could overturn this effect (Figure 6g). Beyond that, A549/DDP and H1975/DDP cell apoptosis was evaluated by flow cytometry, the results exhibited that miR-512-5p could accelerate cell apoptosis, while HMGA1 overexpression could restore the effect of miR-512-5p (Figure 6h). At this moment, transwell assay was conducted to quantify the migration and invasion, it suggested that miR-512-5p inhibited cell migration and invasion; then cell migration and invasion of A549/DDP and H1975/DDP cells were partly reversed after co-transfected with HMGA1 (Figure 6i and j). In addition, tube formation assay was performed to evaluate the effect of HMGA1 on tube formation in A549/DDP and H1975/DDP cells, the results illustrated that miR-512-5p suppressed cells tube formation, but overexpression of HMGA1 could weaken the effect of miR-512-5p on tube formation (Figure 6k). In the end, the protein level of VEGFA was quantified using western blot, the results showed that miR-512-5p reduced the expression of VEGFA, but co-transfected HMGA1 could overturn the trend in A549/DDP and H1975/DDP cells (Figure 6l). In conclusion, overexpression of HMGA1 could restore the effect of miR-512-5p on cell proliferation, migration, invasion, tube formation, and apoptosis.

### 3.7 Anti-miR-512-5p could restore the effect of si-circ-LIMK1#1 in the expression of HMGA1 in DDP-resistant lung adenocarcinoma cells

**Figure 7:**
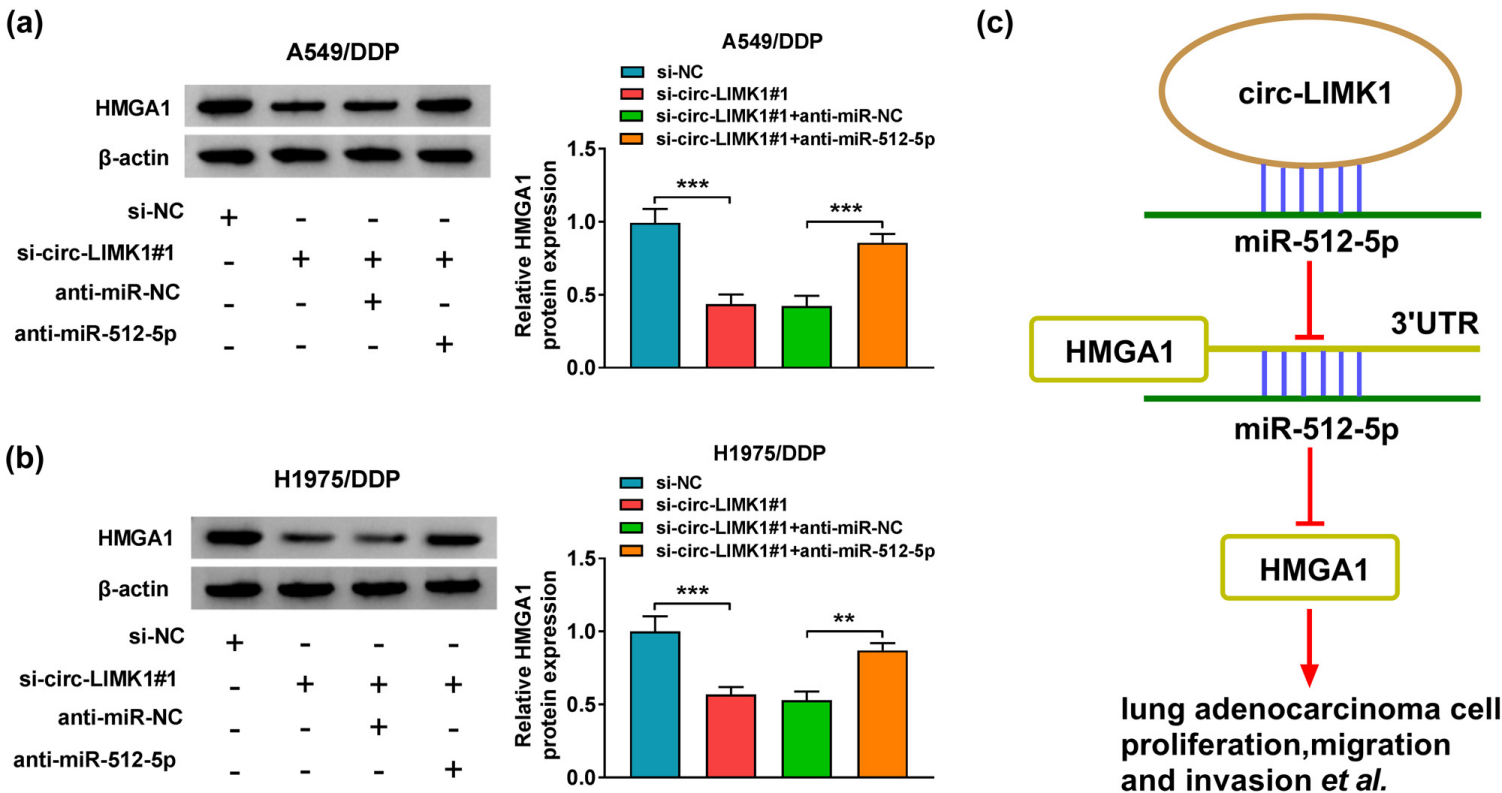
Anti-miR-512-5p could restore the effect of si-circ-LIMK1 in the related progression of lung adenocarcinoma DDP-resistant cells. (a and b) Western blot was used to detect the protein level of HMGA1 in A549/DDP and H1975/DDP cells after transfected with si-NC, si-circ-LIMK1#1, si-circ-LIMK1#1 + anti-miR-NC, or si-circ-LIMK1#1 + miR-512-5p. (c) Functional mechanism diagram of circ-LIMK1, miR-512-5p, and HMGA1. *P < 0.05, **P < 0.01, ***P < 0.001.

The influence of circ-LIMK1 and miR-512-5p on the expression of HMGA1 would be investigated in the next experiment. First, the protein level of HMGA1 was determined, and the results showed that knockdown of circ-LIMK1 could inhibit the expression of HMGA1, but silencing of miR-512-5p could restore the expression of HMGA1 in A549/DDP and H1975/DDP cells (Figure 7a and b). As shown in Figure 7c, circ-LIMK1 could regulate the expression of HMGA1 by targeting miR-512-5p and thus regulate the related processes of DDP-resistant lung adenocarcinoma cells and promote sensitivity of DDP-resistant lung adenocarcinoma cells.

### 3.8 Knockdown circ-LIMK1 could influence tumor growth *in vivo*

**Figure 8:**
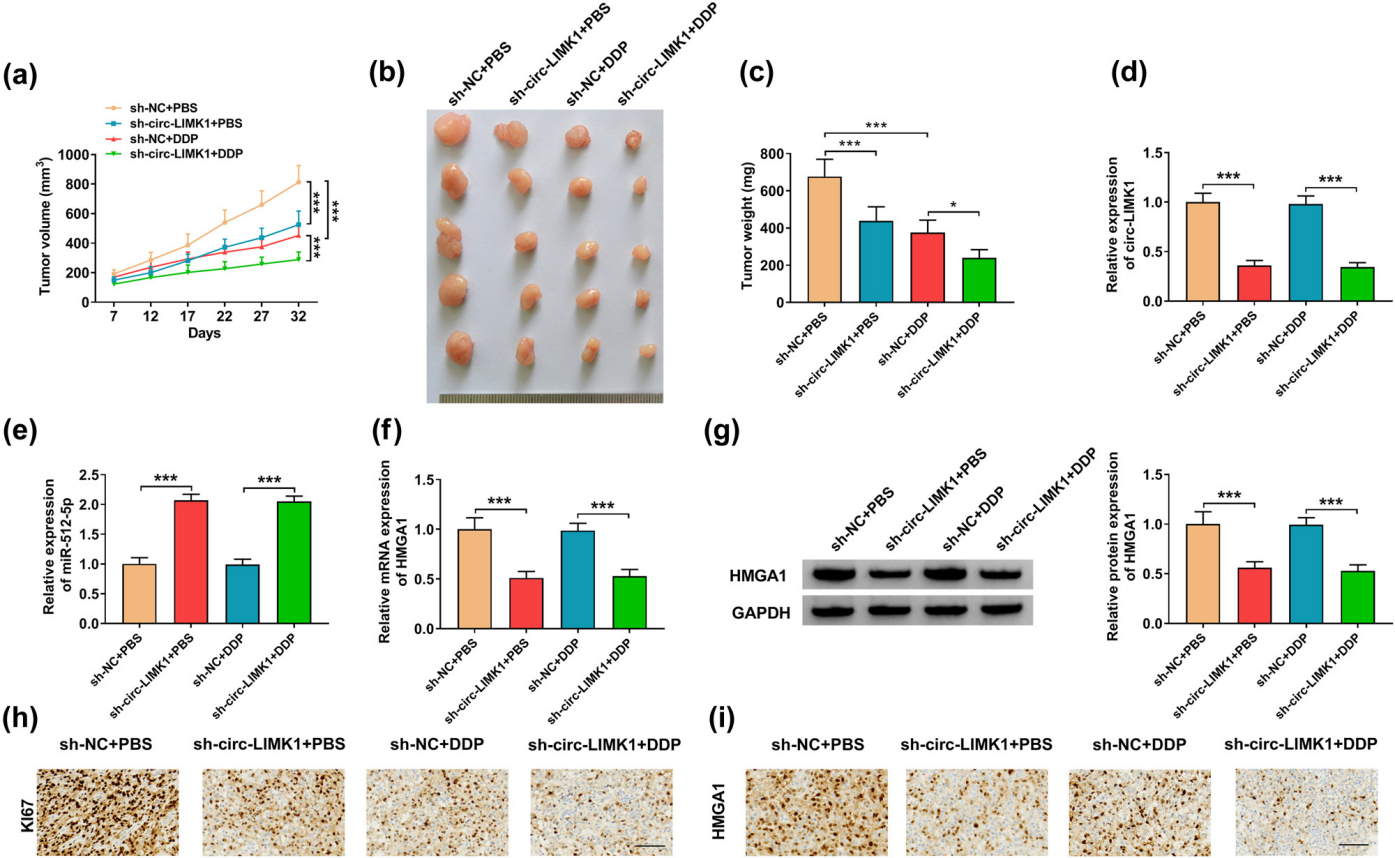
circ-LIMK1 could influence DDP-resistant lung adenocarcinoma tissues. Under sh-NC + PBS, sh-circ-LIMK1 + PBS, sh-NC + DDP, or sh-circ-LIMK1 + DDP conditions. (a–c) The tumor volume and weight were analyzed. (d) RT-qPCR was used to measure the expression of circ-LIMK1. (e) RT-qPCR was used to analyze the expression of miR-512-5p. (f) RT-qPCR was used to measure the mRNA level of HMGA1. (g) Western blot was used to test the protein level of HMGA1. (h and i) IHC was performed to analyze the expression of HMGA1 and Ki67. *P < 0.05, **P < 0.01, ***P < 0.001.

Our study had confirmed the regulatory effect of circ-LIMK1 on lung adenocarcinoma cells at the cellular level, and we would further verify the effect of circ-LIMK1 *in vivo*. H1975/DDP (1 × 10^7^) cells stably expressing sh-NC or sh-circ-LIMK1 were injected subcutaneously into mice, and then analyzed. After 7 days, PBS or DDP was injected every 5 days, and tumor volume was determined. Then tumor weight was detected after the mice were euthanized, the results showed that knockdown circ-LIMK1 could inhibit the volume and weight of tumor, and tumor volume and weight were lower after treatment with DDP compared with PBS (Figure 8a–c). Expressions of circ-LIMK1, miR-512-5p, and HMGA1 mRNA were examined by RT-qPCR, the results suggested that knockdown circ-LIMK1 could reduce the expression of circ-LIMK1 in tumor tissues and the tumor tissues after treatment with DDP (Figure 8d); the expression of miR-512-5p was up-regulated after knockdown of circ-LIMK1 in tumor tissues and the tumor tissues after treatment with DDP (Figure 8e), and the mRNA level of HMGA1 was markedly declined in tumor tissues and the tumor tissues after treatment with DDP (Figure 8f). At the same time, western blot analysis confirmed that knockdown of circ-LIMK1 could decrease the protein level of HMGA1 in tumor tissues and the tumor tissues after treatment with DDP (Figure 8g). Last but not the least, IHC assay provided that knockdown of circ-LIMK1 or DDP treatment could reduce the expression of Ki67 and HMGA1 in tumor tissues, and Ki67 and HMGA1 were lowest after knockdown of circ-LIMK1 and treatment with DDP (Figure 8h and i). In brief, knockdown of circ-LIMK1 could weaken the tumor DDP resistance, DDP did not affect the expression of circ-LIMK1.

## 4 Discussion

Lung adenocarcinoma is one of the cancers with high mortality in the world, and its poor prognosis because of DDP resistance has brought great challenges to its treatment, and also has become one of the problems in the world today [32,33]. Therefore, research related to lung adenocarcinoma has become one of the main contents of the current world research, which will also bring new hope to solve the problem of lung adenocarcinoma DDP resistance in the future.

circRNAs have been reported to play a crucial role in many diseases in recent years [34]. Circ_0001971 could regulate cell proliferation in oral squamous cell carcinoma cells [35]. Circ_0004872 could continuously regulate the progression of gastric cancer [36]. Circ_00016666 inhibited the processes of colorectal cancer [37]. Certainly, many circRNAs were vital in lung adenocarcinoma. For example, circ_0000326 could target miR-338-3p to accelerate the progression of lung adenocarcinoma [38]. Circ_0001715 could be used as a potential biomarker for the diagnosis and prognosis of lung adenocarcinoma [14]. In this study, we explored a new circRNA and found that circ-LIMK1 was up-regulated in tumor tissues, and its expression was significantly boosted in DDP-resistant tumor tissues. The expression level of circ-LIMK1 was higher in A549/DDP and H1975/DDP cells in comparison to their parental cells, which proved that circ-LIMK1 might be involved in DDP resistance of lung adenocarcinoma. In subsequent studies, we found that knockdown of circ-LIMK1 significantly reduced cell proliferation, migration, invasion ability, DDP resistance, and tube formation ability, while significantly increased cell apoptosis. In summary, we preliminarily confirmed that circ-LIMK1 could regulate the development of lung adenocarcinoma and DDP chemoresistance, and on this basis, we speculated that circ-LIMK1 might be a potential target for the diagnosis and prognosis of lung adenocarcinoma.

MiRNAs have been reported to act as tumor suppressor genes or oncogenes in the development of cancers [39]. Of course, miRNAs can work by regulating downstream genes [40]. MiR-195-5p regulated the progression and metastasis of osteosarcoma [41]. MiR-296-5p could be involved in mediating radiosensitivity in colorectal cancer [42]. MiR-195 could play an inhibitory role in the progression of lung adenocarcinoma [43]. In addition, studies showed that miR-512-5p could also play a key role in NSCLC [23]. Therefore, we continued to explore whether circ-LIMK1 could regulate DDP resistance process of lung adenocarcinoma through targeting miR-512-5p. First, after predicting the targeted binding site of circ-LIMK1 and miR-512-5p, we conducted further exploratory experiments. It was found that circ-LIMK1 could target miR-512-5p, and RIP experiment also confirmed the direct binding relationship between circ-LIMK1 and miR-512-5p. Further studies found that miR-512-5p expression was reduced in DDP-resistant lung adenocarcinoma cells, and miR-512-5p absence could overturn the influence of circ-LIMK1 knockdown on the process of DDP resistance of lung adenocarcinoma cells, which also provided important ideas for mechanism research on DDP resistance.

Studies have found that HMGA1 can regulate various physiological processes of NSCLC [28]. Of course, HMGA1 was also involved in the biological process of lung adenocarcinoma [29]. Interestingly, we predicted that miR-512-5p could target HMGA1, and their relationship was further verified by multiple tests. HMGA1 was highly expressed in A549/DDP and H1975/DDP cells or DDP-resistant tumor tissues. The recovery experiment confirmed that HMGA1 overexpression could overturn the effects of miR-512-5p enrichment on the proliferation, migration, invasion, DDP resistance, and apoptosis of A549/DDP and H1975/DDP cells. Based on the above results, we determined that miR-512-5p could regulate DDP resistance and cell phenotypes in lung adenocarcinoma through targeting HMGA1. It is the first time to confirm the mechanism of miR-512-5p/HMGA1 signaling pathway in lung adenocarcinoma.

Further exploration showed that circ-LIMK1 knockdown could inhibit HMGA1 expression, while additional miR-512-5p absence could restore HMGA1 expression. In summary, all the findings confirmed the relationships among circ-LIMK1, miR-512-5p, and HMGA1, as well as their functional mechanism in DDP-resistant lung adenocarcinoma. The construction of mouse transplantation model confirmed that circ-LIMK1 could regulate DDP resistance in lung adenocarcinoma *in vivo*. These results provide a new idea for the mechanism of DDP resistance of lung adenocarcinoma.

## 5 Conclusion

In our study, circ-LIMK1 was highly expressed in DDP-resistant lung adenocarcinoma specimens and cells. Acting as an oncogenic driver, circ-LIMK1 can regulate DDP resistance and tumor development in lung adenocarcinoma by targeting miR-512-5p/HMGA1 pathway. The data further understand the pathogenesis of lung adenocarcinoma.
